# A Novel Framework for Mixed Reality–Based Control of Collaborative Robot: Development Study

**DOI:** 10.2196/36734

**Published:** 2022-05-17

**Authors:** Md Tanzil Shahria, Md Samiul Haque Sunny, Md Ishrak Islam Zarif, Md Mahafuzur Rahaman Khan, Preet Parag Modi, Sheikh Iqbal Ahamed, Mohammad H Rahman

**Affiliations:** 1 Department of Computer Science University of Wisconsin-Milwaukee Milwaukee, WI United States; 2 Department of Computer Science Marquette University Milwaukee, WI United States; 3 Department of Mechanical Engineering University of Wisconsin-Milwaukee Milwaukee, WI United States

**Keywords:** robot framework, mixed reality, collaborative robot, Unity, Windows Mixed Reality, Azure mixed reality services, HoloLens 2

## Abstract

**Background:**

Applications of robotics in daily life are becoming essential by creating new possibilities in different fields, especially in the collaborative environment. The potentials of collaborative robots are tremendous as they can work in the same workspace as humans. A framework employing a top-notch technology for collaborative robots will surely be worthwhile for further research.

**Objective:**

This study aims to present the development of a novel framework for the collaborative robot using mixed reality.

**Methods:**

The framework uses Unity and Unity Hub as a cross-platform gaming engine and project management tool to design the mixed reality interface and digital twin. It also uses the Windows Mixed Reality platform to show digital materials on holographic display and the Azure mixed reality services to capture and expose digital information. Eventually, it uses a holographic device (HoloLens 2) to execute the mixed reality–based collaborative system.

**Results:**

A thorough experiment was conducted to validate the novel framework for mixed reality–based control of a collaborative robot. This framework was successfully applied to implement a collaborative system using a 5–degree of freedom robot (xArm-5) in a mixed reality environment. The framework was stable and worked smoothly throughout the collaborative session. Due to the distributed nature of cloud applications, there is a negligible latency between giving a command and the execution of the physical collaborative robot.

**Conclusions:**

Opportunities for collaborative robots in telerehabilitation and teleoperation are vital as in any other field. The proposed framework was successfully applied in a collaborative session, and it can also be applied in other similar potential applications for robust and more promising performance.

## Introduction

### Background

Robots are becoming friends of humans by impacting and contributing to our daily life in many ways. They can carry out complex or repetitive activities for us, from household to industrial work, medicine, security, and so on [1]. Not only can robots assist in heavy works, but they can also help in education, especially during the COVID-19 pandemic. With the help of artificial intelligence, robots now can act as friends by monitoring behaviors and understanding our likes and dislikes. They are offering various services effectively being used in different industries. Robots can boost the productivity of an industry by performing accurate, precise, fast, consistent, and high-quality work [2]. They can ensure safety by overtaking dangerous tasks in hazardous environments.

Collaborative robots, also known as cobots, are making the situation easier and more productive as they are designed to work in the same workspace as humans [3]. Both the robots and humans work delicately together, and robots assist human coworkers in completing different tasks [4]. This collaboration between humans and robots is changing the industrial production strategy. More industries are shifting to this manufacturing style daily because of flexibility, productivity, safety, reduced risk of injuries, and quality of performance in production [5]. Because of the recent advancement in the collaborative robots’ application in various fields, the size of the global market in this field is growing daily. In 2018, the market value of this industry was around USD 649.1 million and was anticipated to expand to 44.5% by 2025 [6]. Therefore, the potential of collaborative robots, without any doubt, is huge, and more and more research is needed for the refinement of different approaches and applications in this field.

To support the needs of collaborative robots in different potential fields, researchers are working to propose different frameworks for different collaborative robot-based applications. Some researchers followed the agent-based system [7], whereas others also explored compliant control [8-10] and ergonomic aspects–based approach [11]. Still, researchers should consider other state-of-the-art technology-based approaches to designing a strong foundation for the huge potential of collaborative robots. One of the cutting-edge technologies in this era is mixed reality (MR), which blends both the digital and physical worlds to offer a promising solution for various applications [12]. MR merges virtual and augmented reality together, letting us incorporate the real world with digital data. Almost 150 firms in different fields have already adopted MR-based solutions, and it is estimated that by 2025 more than 14.4 million US employees will use smart glasses [13]. The possibility of mixed reality is huge, especially where human interaction is required. Therefore, the application will be promising if the mixed reality can successfully be applied in designing a framework for the collaborative robot.

In this study, a novel framework is proposed for the collaborative robot using mixed reality. Unity, Unity Hub, Windows Mixed Reality (WMR) platform, and Azure mixed reality services are adopted to design the framework and use a holographic device (HoloLens 2) to execute it. The framework can be used in various collaborative applications such as telerehabilitation or teleoperation.

The rest of the manuscript is outlined as follows: first, some recent advancements in this research area are briefly discussed; then, the development of the framework, along with the system architecture and the control of a collaborative robot with mixed reality, is represented; subsequently, one experiment using the framework and result of the experiment is illustrated; and finally, the conclusion of the study is portrayed.

### Related Work

The collaborative robot is one of the potential fields in robotics, and researchers are working on the control system nowadays. Few researchers followed a unified approach by merging an impedance model with a dynamic model with friction and optimizing the assembly [14]. They used the proportional derivative control for the inner loop and impedance control for the outer loop. To evaluate the system’s efficiency, a 6-DOF (Degrees of Freedom) series collaborative robot was used to perform peg-in-hole assembly tasks, and the performance of the system was accurate and flexible. One study proposed that by assessing the mobility of a collaborative robot, the performance of the robot system can be estimated [15]. Most of the available solutions are for a single robot, yet the model was evaluated using 3 automobiles. The model performed more competently than most of the strategies available. Another study represented the humans and robots as coworkers using geometric primitives, attraction vectors, and hypothetical repulsion by computing the distance and relative motion among them [16]. By applying this idea along with the robot’s kinematic representation, the system achieved collision avoidance control by generating a nominal path to cautiously avoid collision with the human while performing the industrial operations. Repulsion-vector-reshaping was also introduced to ensure motion persistence, and the robot performed smoothly and successfully by avoiding collisions.

Rehabilitation robots and assistive robots are two potential applications for collaborative robots. Researchers are working on different approaches using skin surface electromyogram–based signals [17], nonlinear sliding mode control [18], geometric solution [19], and variable transformation for flatness geometric property [20] using collaborative robots to design robots for rehabilitation. Researchers have also followed learning latent actions from task demonstrations [21], reinforcement learning [22], digital image processing [23], and eye tracking–based assistive robot control [24] approaches for collaborative robots, focusing on assistive applications.

One study suggested controlling the momentum of a robot by considering the maximum speed acceptable to secure the safety of human coworkers [25]. The system estimated the allowable top velocity by using a collision model to predict the maximum force during a collision. The system enhanced the functionality and productivity of the collaborative tasks without risking human safety. Few researchers presented a new robotic system for collaborative robots blending mobile manipulators and supernumerary limbs [26]. Their robot could operate autonomously and be connected with humans as additional body parts. Other researchers presented a collaborative system that consists of hardware, software, and operational architecture of a humanoid robot trained with cognitive abilities [27]. The robot could identify the help a human coworker might need, recognize their activities, grasp objects, navigate, and so on. The experimental evaluation demonstrated that the robot performed safely and robustly while conducting collaborative tasks.

Researchers are also exploring framework-based approaches to construct generalized systems for different applications. In one study, researchers proposed an open-source framework for a humanoid robot using cross architecture, which was low-cost in computation [28]. The framework was validated via both simulator and telemetry interface, and the result showed that it could be used to design new algorithms. In another study, researchers presented a framework for a collaborative human-robot environment using a commercial manipulator and their unique control method [10]. The framework included a trajectory planning and safety strategy by exploiting the human worker’s experiences and was evaluated in a factory. In a similar study, a few researchers presented a framework for robot-assisted control in human-robot cooperation for a 7-DoF surgical robot [29]. The framework used manual motion to drive the tooltip, a 3D camera–based method to adjust the workspace, calculation of optimal instrument orientation, and cartesian interpolation to assure safety. In other studies, researchers proposed frameworks for different human-robot collaboration–based applications such as industrial cyber-physical systems [30], interaction in games (ie, Rock-Paper-Scissors) [31], and cooperative assembly duties [32].

The mixed reality–based approaches are becoming popular for different applications among researchers day by day as it has potential in many ways. Researchers used mixed reality–based methods to design an interface for human-robot interaction to teleoperate a robot [33] and a user interface to control teleoperated robotic platforms [34]. Researchers also used these approaches to design various robotic control systems. In one study, researchers developed an interface for human-robot communication using mixed reality for interactive robot manipulation control for mobile platforms [35]. The interface offered tools for robot path planning and visualized it for workers to comprehend robot behavior to ensure safety. The interface was successfully implemented and evaluated on Microsoft HoloLens. In another similar study, researchers used both mixed and virtual reality to design workspace for collaborative robots in the industry [36]. Robotic Operating System and Unity were used to design the system and tested in diverse settings. In another work, researchers presented an interactive control framework for both single and multirobot systems using mixed reality for various applications [37]. The system allowed interaction with robots by focusing on the visualization of their objective, and it could relate to any robots and mixed reality interfaces. The presented framework was evaluated experimentally, and the results verified the framework’s capabilities in the mixed reality system.

## Methods

### Development of a Mixed Reality Framework for Robot Control

To develop the mixed reality–based system from scratch for controlling an assistive robot, some prerequisites and a few prior pieces of knowledge are required. Windows operating system–based computer and windows SDK (software development kit) with visual studio are needed to design the structure. The simplest approach to making mixed reality apps is installing either the Unity or Unreal game engines [38]. However, the same programs may be created for a custom engine using DirectX (Microsoft Corp). DirectX is a high-level interface that allows to directly access low-level functionality. It connects to Windows’ hardware abstraction layer [39]. Unity is one of the most popular real time programming development platforms on the market, with C++-based runtime code and C#-based development scripting [40]. Unity and Unity Hub are used as cross-platform gaming engines and project management tools. The Mixed Reality Toolkit, if Unity is used, may be used for input simulation to test various input interactions, including hand-tracking and eye-tracking input. WMR is a Microsoft platform that debuted with Windows 10. The WMR platform enables developers to create apps that show digital material on holographic and virtual reality displays [41]. The Mixed Reality Feature Tool is needed to configure Unity while developing the framework. Interfacing, generating scenes, importing packages, and adding game objects to a scene all require a basic understanding of Unity. As the Unity scripts are written in C#, some fundamental C# expertise is also required. Any previous knowledge of NoSQL database systems and serverless functionalities will help with the system design. Finally, to implement the application, a holographic device (HoloLens 2) is required. By collecting and revealing digital information within the work setting and surroundings, Azure mixed reality services let people create, learn, and collaborate more efficiently [42]. Azure also helps secure and protect the stored data using 256-bit Advanced Encryption Standard and Transport Layer Security when data are in transit [43]. Azure mixed reality services bring 3D to mobile phones, headphones, and other devices that are not connected to the internet. Azure Remote Rendering and Azure Spatial Anchors are two mixed reality cloud technologies that let developers create captivating, immersive experiences across several platforms. These services enable incorporating spatial awareness into projects when developing 3D training, predictive equipment maintenance, and design review applications, all within the context of users’ environments. The kinematics and dynamics of the assistive robot must be taken into consideration when developing the framework, which is explained below.

#### Assistive Robot

The xArm-5 robot is an end effector robot with 5 DoF, developed by UFactory [44]. To attain a high payload capability (3 kg) and appropriate repeatability of 0.1 mm, this robot is equipped with high-performance harmonic reducers and brushless motors. xArm-5 has a total workspace area of 0.7 meters. The Modbus Remote Terminal Unit protocol is used by xArm-5, and RS-485 communication is required to interact with a position, speed, or force control. These characteristics combine to make the xArm-5 one of the most versatile, high-precision, and multifunction robotic arms on the market. However, xArm-5 is comparatively heavyweight (11.2 kg) due to the big size of the motors; and due to its design features, this robot cannot be folded back during idle time (kinematic constraints). The graphical user interface for xArm-5 is provided by UFactory as xArm Studio and SDK for the languages Python, Robotic Operating System, and C++. Python SDK is used to control the xArm-5 for advanced capabilities in this research.

##### xArm-5 Robot’s Kinematics and Dynamics

During the kinematic analysis, the modified Denavit-Hartenberg (DH) parameters are adopted to specify the xArm configuration of links and joints [45]. On the other hand, the iterative Newton-Euler method was applied for dynamic estimation to assess the joint torques corresponding to each activity of daily living.

##### Forward Kinematics

**Figure 1:**
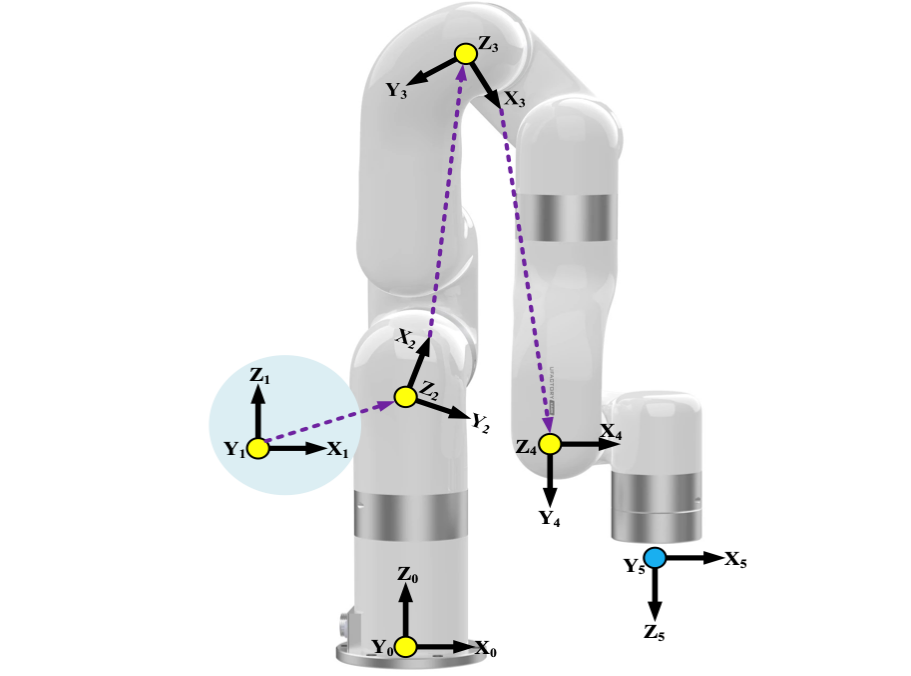
Coordinate frame placement using the modified Denavit-Hartenberg parameters.

The link frame allocation (according to modified DH convention) of the xArm-5 robot is shown in Figure 1, where the yellow dots indicate that the direction is pointing into the viewing surface, cyan dot represents the direction pointing out of the viewing surface, and axis z defines the axis of rotation of each joint. To calculate the forward kinematics, the modified DH parameters corresponding to the link-frame allocation are given in Table 1. Moreover, Table 2 outlines the robot’s link parameters.

**Table 1:** Modified Denavit-Hartenberg parameters of xArm-5 robot.

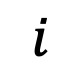	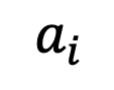	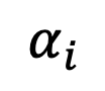	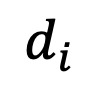	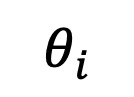
1	0	–π/2	267	0
2	289.4886	0	0	–1.3849
3	351.1587	0	0	2.7331
4	76	–π/2	0	–1.3482
5	0	0	97	0

Here, *ɑ_i_* is the length of the common normal, α_i_ is the angle about common normal, *d_i_* is the offset along the previous z-axis, and 
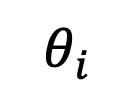
 represents the joint angles. Note that the term 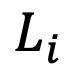 represents the length of the 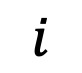 link, and 
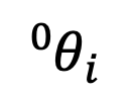
 represents the offset of the angle. The values of those variables are shown in Table 2.

**Table 2:** Dimensional parameters of xArm-5 robots.

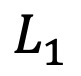	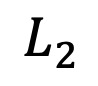	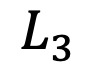	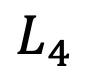	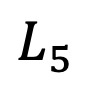	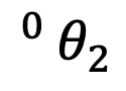	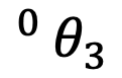	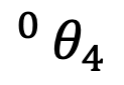
267 mm	284.5 mm	77.5 mm	342.5 mm	76 mm	–1.3849 rad	2.7331 rad	–1.3482 rad

The general form Homogeneous Transformation Matrix that relates two successive coordinate frames is represented by Equation (1).




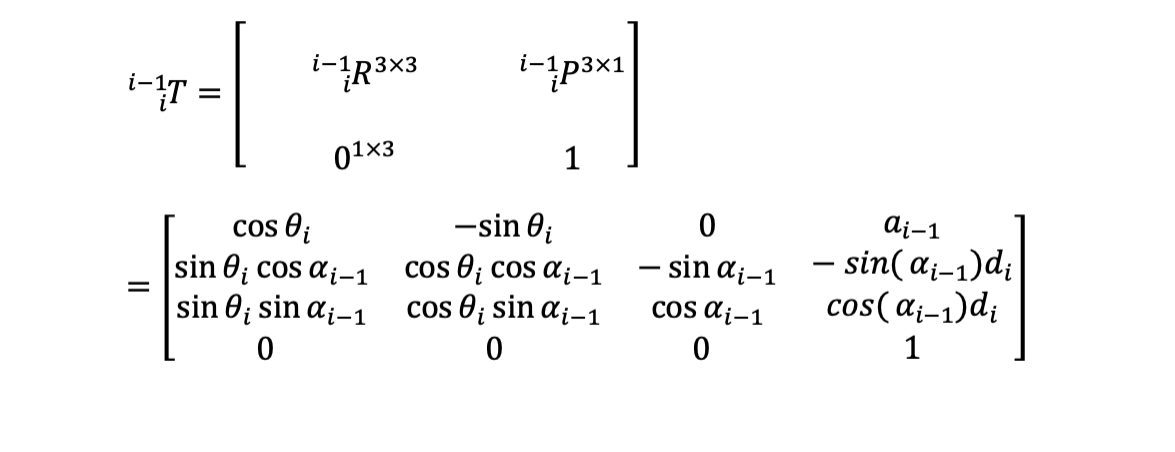




Where 
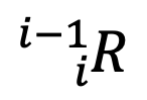
 is the rotation matrix that represents the frame 
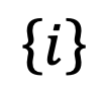
 in relation to frame 
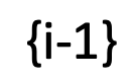
, and 
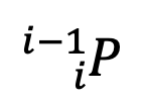
 is the vector that indicates the location of the origin of the frame 
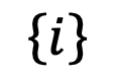
 with respect to the frame 
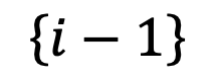
.

Moreover, 
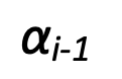
 is the link twist, 
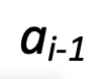
 corresponds to link length, *d_i_* stands for link offset, and *θ_i_* is the joint angle (radian) of the xArm5 Robot. The individual homogeneous transfer matrices that relate two successive frames of the xArm robot (Figure 1) are derived using Equation 1 and are given in Multimedia Appendix 1. The homogenous transformation matrix that relates frame {5} to frame {0} can be obtained by multiplying individual transformation matrices as expressed in Equation (2).




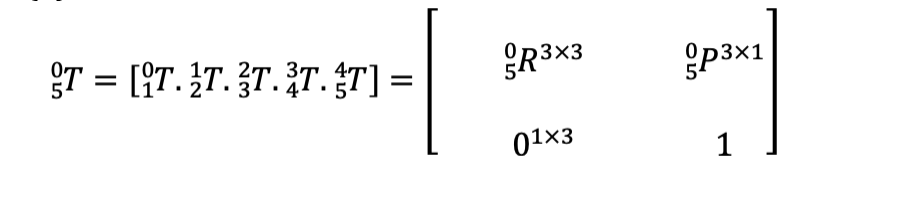




The single transformation matrix found from Equation (2) represents the reference frame’s positions and orientations attached to the end effector with respect to the base reference frame {0}.

##### Dynamics of the xArm-5 Robot

The dynamic equation of the xARm5 Robot derived from the Newton-Euler formulation can be written in the following form:




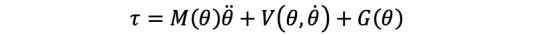




Where 
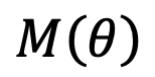
 is the 5×5 mass matrix of the manipulator, 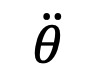 are the 5×1 acceleration vector, 
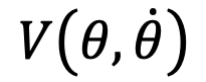
 is the 5×1 vector of centrifugal and Coriolis terms, and *G(θ)* is the 5×1 vector of gravity terms. Table 3 summarizes the mass/inertia parameters of the xArm-5 robot, and joint torques for the xArm-5 were calculated using Equation 3. Moreover, Table 4 presents the range of motion of each joint.

**Table 3:** Inertial parameters for each link of xArm-5 robot.

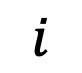	Mass (kg)	Center of mass (mm)
Link 1	2.177	[0.15, 27.24, –13.57]
Link 2	2.011	[36.7, –220.9, 33.56]
Link 3	2.01	[68.34, 223.66, 1.1]
Link 4	1.206	[63.87, 29.3, 3.5]
Link 5	0.17	[0, –6.77, –10.98]

**Table 4:** Range of motion.

Joint			Working range
**Joint number, deg**			
	Joint 1	±360	
	Joint 2	–118~120	
	Joint 3	–225~11	
	Joint 4	±360	
	Joint 5	–97~180	
Maximum speed, deg/s			180

##### xArm-5’s Control Architecture

**Figure 2:**
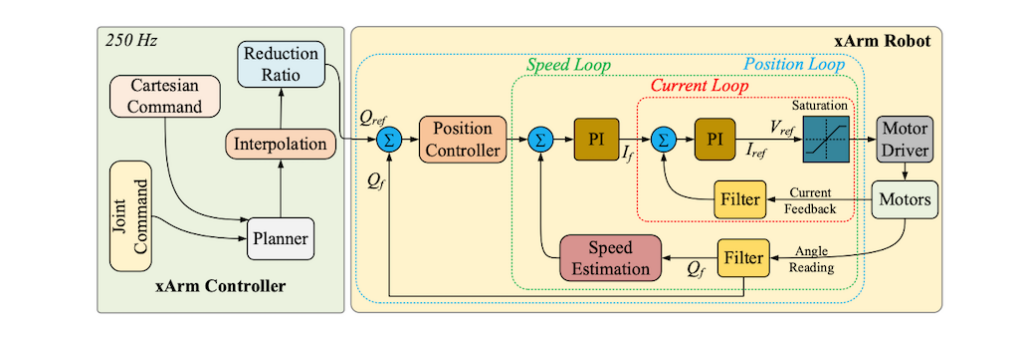
Control architecture of the system. I_f_: filtered current; I_ref_: reference current; PI: proportional integral; Q_f_: filtered joint angle; Q_ref_: reference joint angle; V_ref_: reference voltage.

Figure 2 illustrates the control architecture for the xArm-5 robot. The xArm controller generates two commands, the joints’ torque and the cartesian, and updates the torque commands every 4 ms to execute in the xArm-5 controller. The torque commands are transformed to motor currents and finally to reference voltage for the motor drivers. The proportional integral controller is employed to acknowledge the real time control of the system. It also guarantees that the suitable control torque commands are transmitted to the joints and the reference voltage commands for the drivers. It also minimizes the differences between the desired and the measured currents.

#### System Architecture

**Figure 3:**
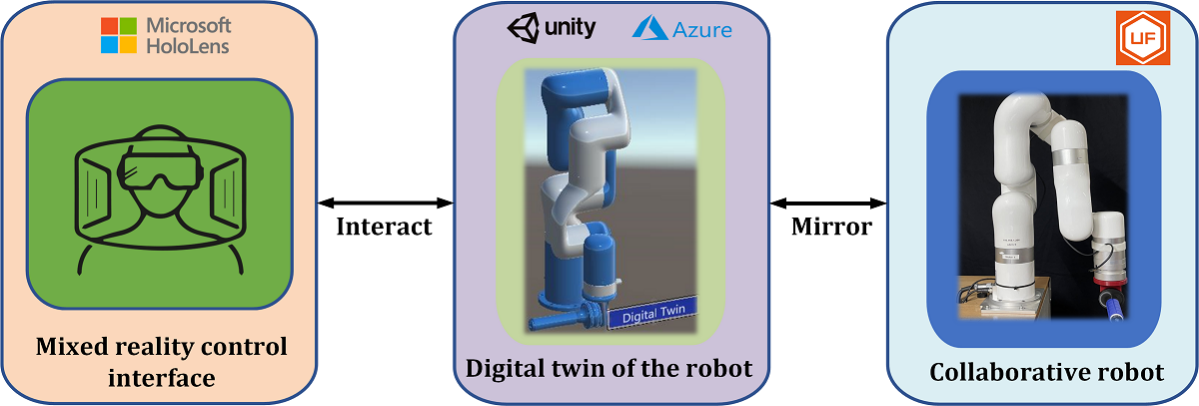
Data flow of the connected system.

This section discusses the life cycle of a collaborative session involving a collaborative robot in a mixed reality environment. The user launches the application from their Mixed Reality headset, which is the application’s access point (HoloLens 2). With the suitable digital twin and user, the provider can construct a collaborative session room. The user, as well as any allowed onlookers, can now join the collaboration session. Even though they are in separate places, all users in the collaboration session, including the host user (provider), are now in a digital collaboration session and communicating with the identical digital twin. Depending on the different needs, the provider can regulate the digital twin in several ways. Figure 3 shows the data flow of the connected system.

**Figure 4:**
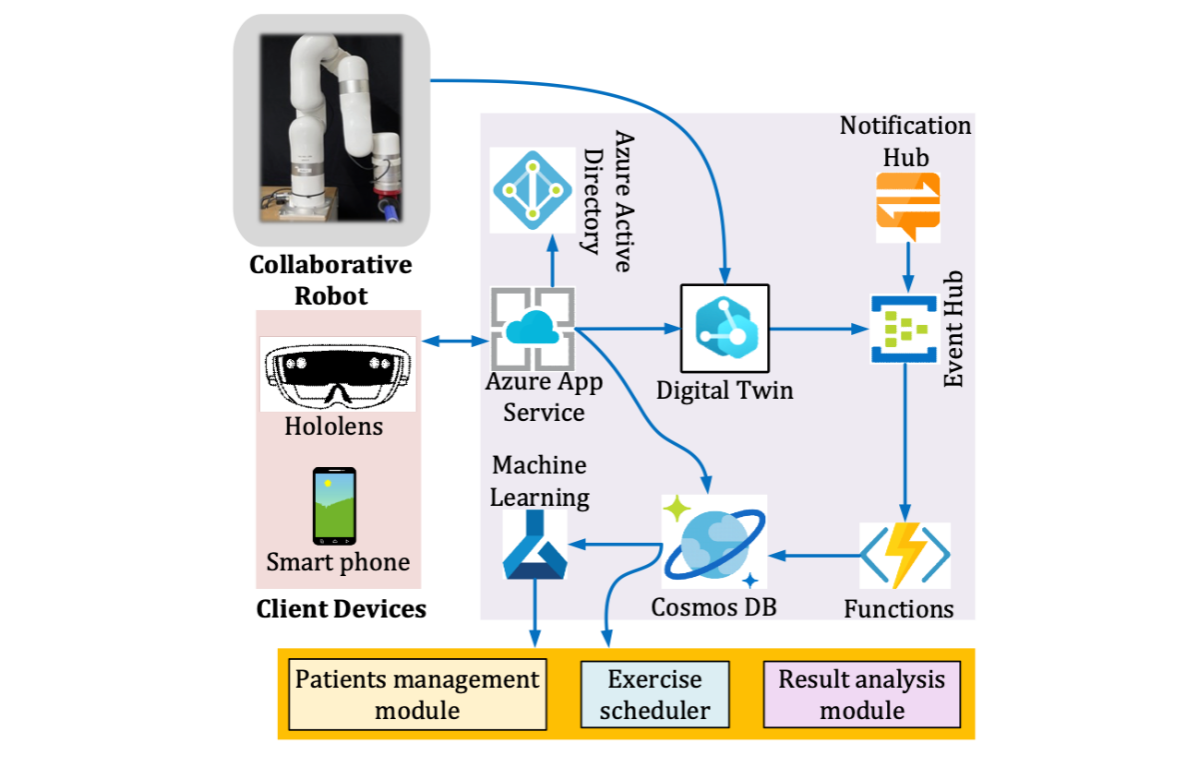
Detailed system architecture.

Due to the distributed nature of cloud applications, the system can send a command to the digital twin, which the collaborative robot physically executes. All commands require a controller that runs on the robot’s local network. WebSocket is a computer communications protocol that allows full-duplex communication channels over a single Transmission Control Protocol connection, which manages all device connections and interactions [46]. Users of the mixed reality collaborative session can now use high-level commands to control the collaborative robot. If the provider wants to control the robot to the desired path, it will be confined to moving along a fixed course and at varying distances along that trajectory. The user can provide input to the provider during this procedure, and key data such as the joint parameters and torques are collected and sent to the cloud. For example, using the Mixed Reality headset, the user can play interactive games that target specific muscles or motions to create a sense of confidence and accomplishment in their physical development. Thus, the entire quality of the therapy is improved. Internet of Things data can be stored and retrieved using the Azure cloud platform. Every collaborative robot sends telemetry data to a cloud platform, where it is stored indefinitely. In the case of teletherapy, relevant parameters such as patient range of motion and resistance to motion during various exercises are pushed to the cloud during each rehabilitation session. These parameters are used by the Azure cloud platform to support machine learning approaches that can adaptively develop appropriate rehabilitation strategies for each user. Figure 4 shows the details of the system architecture.

At first, in the event sequence of the mixed reality environment, the user launches the app on the client device and connects with Azure App Service via WebSocket. Then, the user is authenticated via Azure Active Directory. Subsequently, the user can select a digital twin model they wish to interact with. The App Service will retrieve assets corresponding to the selected digital twin, including 3D models and data. Next, the App Service provides a user with their requested data. The Digital Twin pushes incoming data to the Event Hub when the system is running, which fires a serverless function on the Azure cloud server. The serverless function updates database values. At the final step of data flow, some machine learning models will be deployed, which will use historical users’ data and real time collaboration metrics for future automated result analysis.

#### Control of a Collaborative Robot Through the Mixed Reality Environment

The Azure cloud platform enables multiple users to join a shared collaboration session. In this collaboration session, users can visualize and interact with one digital twin. The digital twin will move in tandem with the physical robot and mirror its movements. Users in the collaboration session can additionally send high-level commands to control the digital twin.

**Figure 5:**
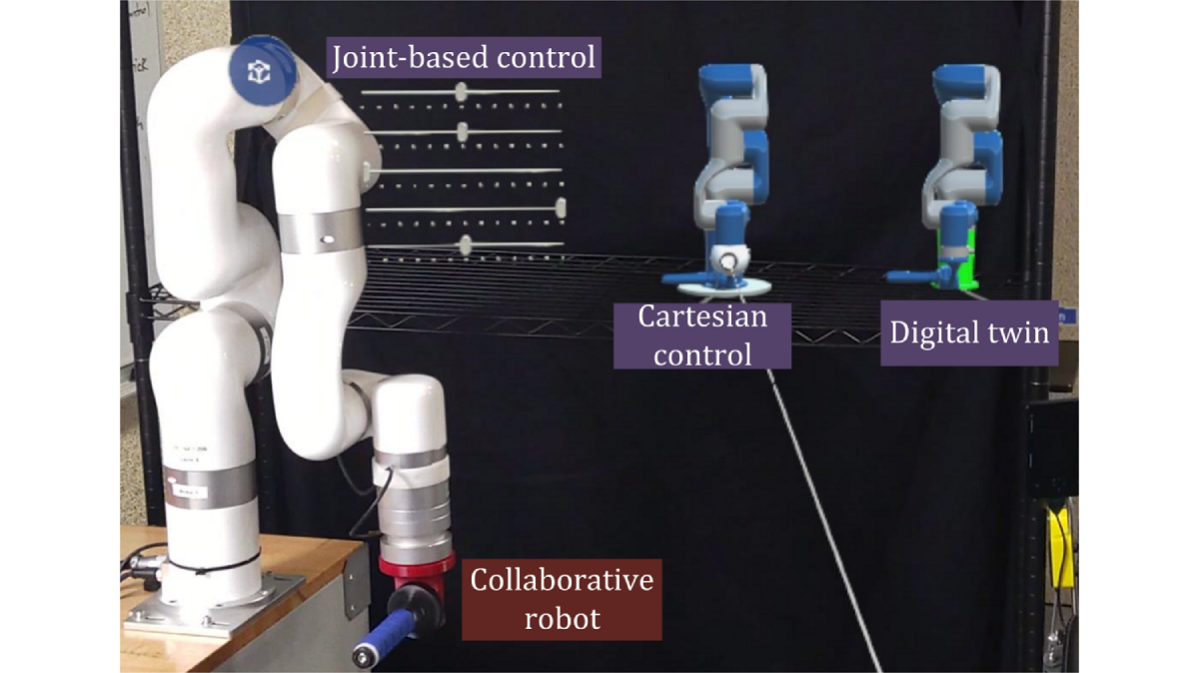
Mixed reality interface.

Figure 5 shows the mixed reality user interface of the proposed framework. During the collaboration session, a user can control a collaborative robot in several ways, such as joint angle control, cartesian control, and preplanned trajectory control. The virtual sliders are used in joint-based control. On the other hand, in cartesian mode end effector is controlled by moving the virtual end effector in a mixed reality environment. Furthermore, the provider can set a desired path to follow for a collaborative robot in the preplanned trajectory control mode.

### Ethical Considerations

The researchers involved in the project took part in the experiments to demonstrate the proof of concept of the teleoperation using the proposed framework. Therefore, ethical approval is not required for this study. The project did not focus on any intervention development or intervention efficacy testing; hence, we did not recruit any participants and did not seek ethics approval for this project.

## Results

**Figure 6:**
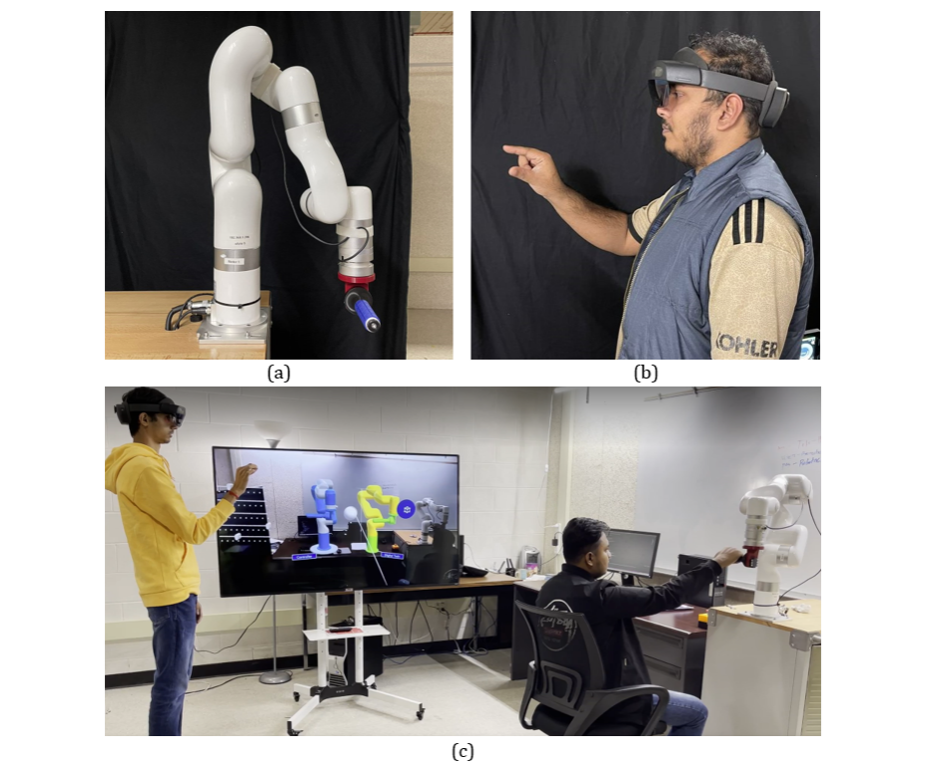
Experimental setup for the proposed collaborative robot framework: (a) xArm-5 Robot as the collaborative robot; (b) user with HoloLens 2 headset; and (c) collaborative session via the mixed reality–based framework (Multimedia Appendix 2).

To validate the proposed framework, an end effector type 5 DoF assistive robot is mounted on a table (Figure 6a). The purpose of this assistive robot is to give therapy with a pretrained trajectory and by a practitioner. The designed app, which contains the mixed reality interface, is deployed to the HoloLens 2 to control the assistive robot. A practitioner can wear the HoloLens 2 and control the therapeutic sessions remotely in a mixed reality environment (Figure 6b). Figure 6c depicts a collaboration session between a practitioner and a patient where the robot can be controlled and monitored remotely in a mixed reality environment using the proposed framework.

**Figure 7:**
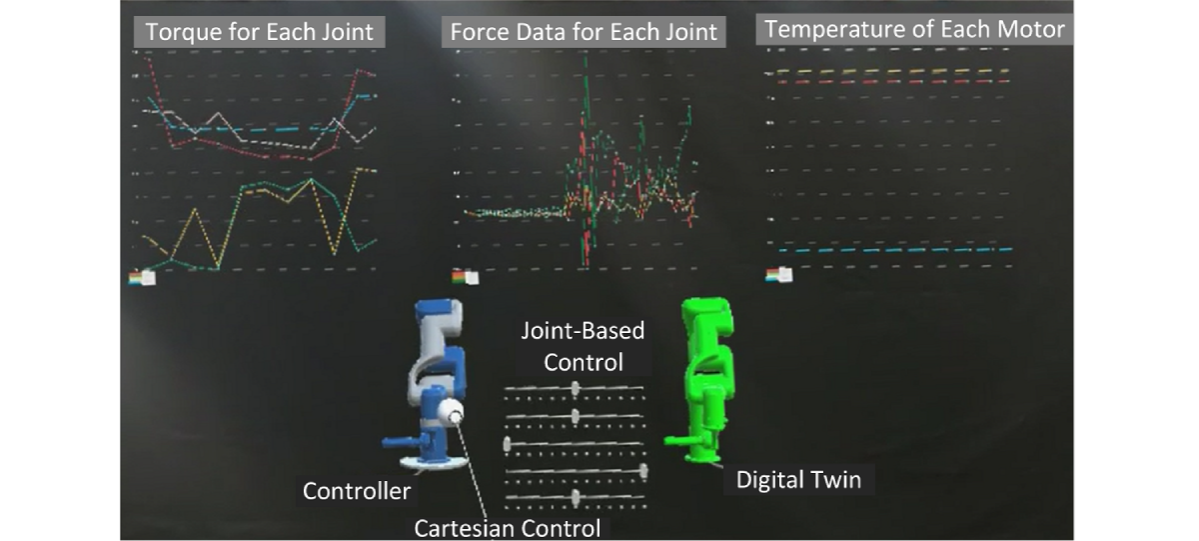
Visualizing the key metrics in the interactive mixed reality environment.

The system provides an overall cross-platform rehabilitation experience for its users. A clinician can remotely assist a patient in both active and passive rehabilitation therapy via a shared mixed reality–based collaboration session. The rehabilitation robot that exists locally for the patient has a digital twin that lives on the Azure cloud platform. A therapist or clinician can interact with this virtual digital twin and use it to assist with rehabilitation therapy. Figure 7 shows a mixed reality interface that visualizes robot data such as torque and temperature for each joint, as well as force sensor data. In this manner, the therapist can visualize the key metrics that estimate the patients’ improvement, such as range of motion and resistance. They can use these data to recommend optimal rehabilitation plans for a patient. Users in the collaboration session can summon data visualizations of both real time and historical data.

## Discussion

Framework for collaborative robots has many potential applications in industry and telehealth such as teleoperation and telerehabilitation. Especially, a pandemic situation such as COVID-19 has affected all aspects of health care and gave the realization of the need for telehealth, which can help health care workers and patients to protect themselves from the risk of disease transmission. Telehealth can also offer personalized rehabilitation programs, real time control, continuous interaction with doctors, negligible waiting time, and so on. Using the proposed framework, it will be handy to implement different systems for teleoperation. While the proposed mixed reality–based framework promises a stable control system for collaborative robots, there are some limitations to it [47-49]. To use the framework, there should be a continuous and stable connection. The system becomes unstable and inoperable if the connection is lost. Security of personal health data is also a concern. Furthermore, the system is expensive compared to other regular rehabilitation, as a holographic device and a collaborative robot is needed for this [50]. It is worth mentioning that a holographic device such as HoloLens 2 should not be worn for extended periods of time. Possible side effects of HoloLens include headache, dizziness, or loss of balance [50,51]. It is important to use these tools responsibly to achieve the maximum benefit from the services they provide. To improve the framework, in the future, the focus will be given to stable communication, personalized rehabilitation program, and real time control and monitoring by the expert.

### Conclusion

Collaborative robots and their applications have a magnificent impact in the rehabilitation and teleoperation fields. A framework for collaborative robots is very much needed to meet the demands of collaborative robots. The proposed mixed reality–based framework for collaborative robots can be used for different telehealth applications such as teleoperation and telerehabilitation and can guide other researchers to conduct further studies for the advancement of humans. Several state-of-the-art technologies were used while developing the framework, including Unity, WMR platform, Azure mixed reality services, and HoloLens 2. The framework is validated by conducting a comprehensive, collaborative experiment using a 5-DoF collaborative robot (xArm-5) in a mixed reality environment. In the future, the study will continue with mixed reality–based applications for collaborative robots. Further research will be conducted on telerehabilitation and teleoperation to design a more robust and stable framework for further advancement.

## Appendix

Multimedia Appendix 1 Individual homogeneous transfer matrix.

Multimedia Appendix 2 Experiment of collaborative session via mixed reality–based framework.

## Abbreviations

DH: Denavit-Hartenberg
DOF: Degrees of Freedom
MR: Mixed Reality
SDK: software development kit
WMR: Windows Mixed Reality
