# Supplementary material for: A Novel Framework for Mixed Reality–Based Control of Collaborative Robot: Development Study
Source: JMIR Biomed Eng. 2022 May 17;7(1):e36734. doi: 10.2196/36734 (PMC11041473; doi:10.2196/36734)
Supplement: Multimedia Appendix 1 [file biomedeng_v7i1e36734_app1.docx]

**Multimedia Appendix 1**. Individual homogeneous transfer matrix.

| ${}_{1}^{0}T=\left[ \begin{matrix} \cos\theta_{1} & -sin \theta_{1} & 0 & 0 \\ \sin\theta_{1} & \cos\theta_{1} & 0 & 0 \\ 0 & 0 & 1 & L_{1} \\ 0 & 0 & 0 & 1 \end{matrix} \right]$ | **(1)** |
| --- | --- |

| ${}_{2}^{1}T=\left[ \begin{matrix} \cos{(\theta}_{2}+{^{\circ}\theta}_{2}) & -sin {(\theta}_{2}+{^{\circ}\theta}_{2}) & 0 & 0 \\ 0 & 0 & 1 & 0 \\ -sin {(\theta}_{2}+{^{\circ}\theta}_{2}) & -\cos{(\theta}_{2}+{^{\circ}\theta}_{2}) & 0 & 0 \\ 0 & 0 & 0 & 1 \end{matrix} \right]$ | **(2)** |
| --- | --- |

| ${}_{3}^{2}T=\left[ \begin{matrix} \cos{(\theta}_{3}+{^{\circ}\theta}_{3}) & -sin {(\theta}_{3}+{^{\circ}\theta}_{3}) & 0 & L_{2} \\ \sin{(\theta}_{3}+{^{\circ}\theta}_{3}) & \cos{(\theta}_{3}+{^{\circ}\theta}_{3}) & 0 & 0 \\ 0 & 0 & 1 & 0 \\ 0 & 0 & 0 & 1 \end{matrix} \right]$ | **(3)** |
| --- | --- |

| ${}_{4}^{3}T=\left[ \begin{matrix} \cos\left( \theta_{4}+{^{\circ}\theta}_{4} \right) & -sin \left( \theta_{4}+{^{\circ}\theta}_{4} \right) & 0 & L_{3} \\ \sin\left( \theta_{4}+{^{\circ}\theta}_{4} \right) & \cos\left( \theta_{4}+{^{\circ}\theta}_{4} \right) & 0 & 0 \\ 0 & 0 & 1 & 0 \\ 0 & 0 & 0 & 1 \end{matrix} \right]$ | **(4)** |
| --- | --- |

| ${}_{5}^{4}T=\left[ \begin{matrix} \cos\theta_{5} & -sin \theta_{5} & 0 & L_{4} \\ 0 & 0 & 1 & L_{5} \\ -sin \theta_{5} & \cos\theta_{5} & 0 & 0 \\ 0 & 0 & 0 & 1 \end{matrix} \right]$ | **(5)** |
| --- | --- |
